# Treatment Interruption and Regimen Change in Firstgeneration versus Second-generation Tyrosine Kinase Inhibitors used as First-line Therapy for Chronic Myeloid Leukemia

**DOI:** 10.36469/9899

**Published:** 2015-04-27

**Authors:** Melea A. Ward, Gang Fang, Kristy L. Richards, Christine M. Walko, Stephanie R. Earnshaw, Laura E. Happe, Susan J. Blalock

**Affiliations:** 1 University of North Carolina, Division of Pharmaceutical Outcomes and Policy, Chapel Hill, NC; 2 Lineberger Comprehensive Cancer Center, Division of Hematology/Oncology, Chapel Hill, NC; 3 University of North Carolina, Division of Pharmacotherapy and Experimental Therapeutics, Chapel Hill, NC; DeBartolo Family Personalized Medicine Institute, H. Lee Moffitt Cancer Center and Research Institute, Tampa, Florida; 4 RTI Health Solutions, RTI International, Durham, NC; 5 Humana, Inc., Louisville, KY

**Keywords:** oncology, hematology, treatment interruption, tyrosine kinase inhibitors, chronic myeloid leukemia

## Abstract

**Background:** Research has shown that treatment interruptions are associated with worse failure-free survival in chronic myeloid leukemia (CML); however they are commonly used in clinical trials to manage adverse events.

**Objectives:** This study assessed the comparative rates of treatment interruption and regimen change between patients initiating first-line therapy with a first-generation tyrosine kinase inhibitor (1GTKI) imatinib versus second-generation TKI (2GTKI), dasatinib or nilotinib, for the treatment of CML in clinical practice.

**Methods:** This was a retrospective cohort study using the Humana Research Database. Patients with CML who were between the ages of 18 and 89 and newly initiated 1GTKI or 2GTKI therapy between June 1, 2010 and December 31, 2011 were included. Treatment interruption and regimen change were compared using multivariable Cox proportional hazard regression models. Treatment interruption was defined as a gap in any TKI pharmacy claim that was longer than an allowable refill gap plus days’ supply from the previous TKI medication claim. Regimen change was defined as 1) a prescription claim for a different TKI therapy, or 2) increase in dose for the same medication.

**Results:** 368 patients met the inclusion criteria: 1GTKI n=237, 2GTKI n=131. Patients initiating therapy with a 2GTKI had a 48% higher risk of treatment interruption versus patients initiating therapy with a 1GTKI (hazard ratio=1.48, 95% confidence interval 1.08-2.02). The time to treatment interruption was significantly longer in patients initiating therapy with a 1GTKI. Approximately 19% of patients had a regimen change, but there were no differences in rates of regimen changes between the two generations.

**Conclusions:** In this study from a large single health plan population, treatment interruptions were more common among patients initiating therapy with a 2GTKI, yet regimen change rates did not vary by generation of TKI. Future research should assess reasons for treatment interruption and investigate these associations in other populations.

### BACKGROUND

Tyrosine kinase inhibitors (TKIs) are the standard of care in treating chronic phase chronic myeloid leukemia (CML). These agents have demonstrated overall survival benefits exceeding 93% at 3 years in clinical trials.[1,2] Imatinib was the first TKI approved for treatment of chronic phase CML in the United States (US) in 2002 and is considered a first-generation TKI (1GTKI). Second-generation TKIs (2GTKIs) approved for first-line therapy in the US include nilotinib and dasatinib.

Although first- and second-generation TKIs offer high survival and are generally well tolerated, treatment interruptions and dose modifications are commonly used to manage adverse events.[1-6] In a randomized Phase III comparative trial over 12 months, 59%-66% (depending on dose) of nilotinib patients and 52% of imatinib patients had a dose reduction or treatment interruption.[6] Based on the 12-month data of a randomized Phase III comparative trial, 52% of dasatinib patients and 35% of imatinib patients had a treatment interruption with the median duration of the first dose interruption due to toxicity occurring at 13 to 14 days.[7]

A study conducted at MD Anderson Cancer Center revealed that treatment interruptions might be associated with adverse clinical consequences.[8] The study was a retrospective review of records from 343 adult patients enrolled in open label Phase I-III trials of dasatinib or nilotinib between 2004 and 2008. Overall, 63% of patients required treatment interruptions and/or dose reduction at least once during therapy. Dose reductions/interruptions were associated with worse failure-free survival than in the overall patient population ($P<0.001$). However, there was no association between treatment interruptions and overall survival.

Treatment interruption patterns from clinical trials may not be reflective of clinical practice utilization. Two observational studies have examined imatinib treatment interruptions. The first was a retrospective analysis of managed care data that reported 31% of 267 patients taking imatinib for CML experienced treatment interruptions.[9] The second was a retrospective chart review of 177 patients treated with imatinib for CML.[10] Rates of treatment interruption, discontinuation, and switching to another therapy were 16%, 24%, and 23%, respectively. In clinical practice, a therapy change or switching therapies is often indicative of treatment failure or intolerance. For example, a patient who has lost a complete cytogenetic response (CCyR) or a complete hematologic response (i.e., secondary resistance) should change therapy rapidly.[11] A delayed treatment change in a patient who has lost cytogenetic response can decrease the probability of event-free survival.[12]

Although these two studies provide real-world evidence of treatment patterns with imatinib, they fail to report comparative data for 2GTKIs. We recently described the association between initiating 2GTKI versus 1GTKI and medication adherence, health services utilization, and healthcare costs.[13] Comparative evidence is important for TKI therapy choice, as imatinib, nilotinib, and dasatinib are all recommended as first-line treatment options with a category 1 level of evidence by the National Comprehensive Cancer Network (NCCN) Guidelines for CML.[14] Additionally, although 2GTKIs have demonstrated superior clinical outcomes,[3,6] imatinib may be the therapy of choice in some cases, as it is effective in approximately two-thirds of patients and is substantially less expensive than the 2GTKIs.[15-16] Comparative data from clinical practice on treatment interruptions, which are associated with worse failure-free survival, will provide oncologists with additional data to support their therapeutic selections. Here we describe the association between newly initiating therapy with a 2GTKI (nilotinib or dasatinib) versus 1GTKI (imatinib) and 1) treatment interruption and 2) regimen change.

### METHODS

#### Data Source

This retrospective observational cohort study was conducted using data from the Humana Research Database between February 1, 2010 and December 31, 2012. The Humana Research Database includes pharmacy claims, medical claims, and enrollment data for over 1.5 million commercial fully-insured members, 1.9 million Medicare Advantage members, and 2.4 million Medicare Part D members from all 50 states. Pharmacy claims data contain adjudication information for prescription medications, including drug name, dosage, quantity, day supply, and date of fill. Enrollment data contain information on demographics and coverage start and end dates. Medical claims were not used for this study. Plan paid and patient paid amounts are available for all claims. The University of North Carolina Institutional Review Board determined that this study was exempt from review.

#### Sample Selection

Adult patients newly initiating a TKI as first-line therapy for CML during the study intake period between June 1, 2010 and December 31, 2011 were eligible for inclusion. The intake period was based on the FDA approval of first-line indication for the 2GTKIs in June and October of 2010, permitting up to a 1-year of follow-up before the study end date of December 31, 2012 (Figure 1). The index date was defined as the first pharmacy claim for imatinib, nilotinib, or dasatinib during the intake period. A 4 month baseline period with no pharmacy claims for TKI therapy prior to the index date was used to define an incident user of a TKI. This baseline period allowed for a 30 day refill gap for patients who may have filled a prescription for a 90-day supply. Patients were followed for up to 12 months after the index date, which was defined as the follow-up period.

**Figure 1. Study Design**

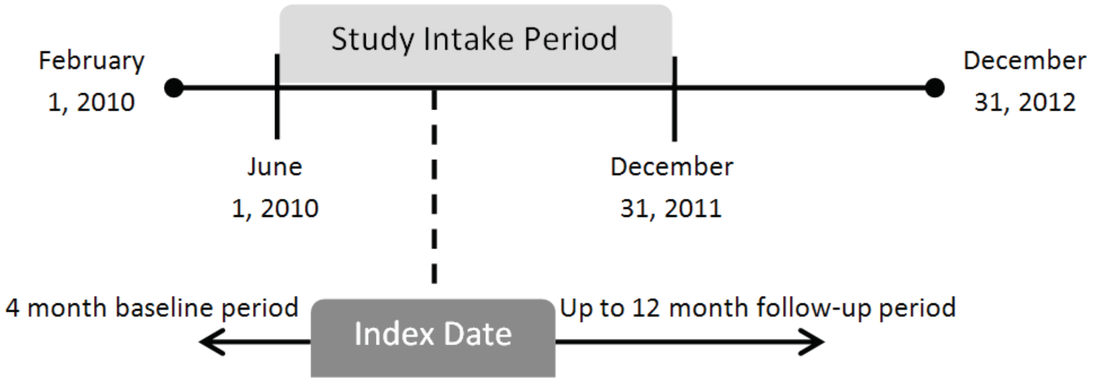

The index date was defined as the first pharmacy claim for imatinib, nilotinib, or dasatinib during the study intake period. A 4 month baseline period with no pharmacy claims for TKI therapy prior to the index date was used to define an incident user of a TKI. Patients were followed for up to 12 months after the index date, which was defined as the follow-up period.

Eligible patients were included in the study sample if they (1) had at least one claim for imatinib, nilotinib, or dasatinib during the intake period; (2) were new users to TKI therapy defined as having no claim for a TKI therapy during the baseline period; (3) were continuously enrolled in a Humana plan with prescription drug coverage throughout the baseline period; (4) were between the ages of 18 and 89 years on the index date; and (5) had a diagnosis for CML, determined via manual review of prior authorization forms, at the time the TKI

therapy was initiated. Included patients were categorized into two mutually exclusive treatment groups based on receipt of a 1GTKI (imatinib) or 2GTKI (dasatinib or nilotinib) as their index medication.

Baseline characteristics were reported for both TKI treatment groups. Multivariate logistic regression was used to assess the factors associated with initiating therapy with a 2GTKI versus 1GTKI. The following covariates were included in the models: patient demographic characteristics (age, gender, plan type, region, low income subsidy, dual eligibility status), clinical characteristics (RxRisk-V score, phase of CML disease using starting dose of TKI as a proxy, flu or pneumonia vaccination, medication count), and provider characteristics (age, gender, specialty, provider practice setting). The RxRisk-V is a prescription claims-based comorbidity index originally developed as an enhancement of the RxRisk assessment instrument for use in the Veterans Health Administration population.[17-19] Each TKI therapy has a dose that suggests suboptimal or accelerated phase disease. For phase of CML disease, patients' starting dose of TKI therapy at the time of initiation was assessed and used as a proxy to distinguish chronic phase dosing versus suboptimal or accelerated phase/blast crisis dosing. The dose was calculated as the strength of TKI therapy dispensed, multiplied by the quantity filled, divided by the days' supply.

#### Treatment Patterns and Statistical Analyses

The two treatment patterns assessed in this study were treatment interruption and regimen change. Treatment interruption was defined as a gap in any TKI pharmacy claim that is longer than an allowable refill gap plus remaining days' supply from the previous TKI medication claim. An allowable refill gap of 30 days was used, based upon the methods used in a previous study of treatment interruptions with imatinib.[9] The percentage of patients with a treatment interruption was reported for both treatment groups. For patients with a treatment interruption, medication behavior following the treatment interruption was reported as re-initiation, medication change, discontinuation, or disenrollment.

Regimen change was defined as 1) a prescription claim for a different TKI therapy than the patient initiated (imatinib, dasatinib, nilotinib, or bosutinib), or 2) increase in dose for the same TKI therapy initiated during the follow-up period. The percentage of patients with a regimen change was reported for both treatment groups.

Patients were followed until the date of the occurrence of treatment interruption or regimen change, end of enrollment, or the end of the 12-month follow-up period, whichever came first. Because patients had different lengths of follow-up, Cox proportional hazard regression models were used to measure the association between TKI treatment group and treatment interruption and regimen change. The following covariates were included in the models: patient demographic characteristics (age, gender, plan type, region, low income subsidy, dual eligibility status), clinical characteristics (RxRisk-V score, phase of CML disease using starting dose of TKI as a proxy, flu or pneumonia vaccination, medication count), and provider characteristics (age, gender, specialty, provider practice setting). Additionally, two sensitivity analyses were conducted. The first limited the population to those with chronic phase disease as this is the most clinically relevant data for the treating physician to help in the selection of first-line treatment (imatinib versus 2GTKIs), because most patients in advanced phase will directly receive 2GTKIs. The second sensitivity analysis was completed to censor for death as a competing risk. Therefore, in this sensitivity analysis, time to event was defined as time elapsed between first TKI therapy fill (i.e. index date) and date of treatment interruption, end of enrollment, date of death, or the end of the study period, whichever came first.

All analyses were performed using SAS Enterprise, version 4.3 (SAS Institute, Cary, NC, US). Alpha was set a priori at 0.05 for all statistical tests.

## RESULTS

### Sample Characteristics and Treatment Choice

There were 2,369 unique patients treated with a TKI between June 1, 2010 and December 31, 2011 (Figure 2). Of these, 368 (1GTKI n=237, 2GTKI n=131) patients met the eligibility criteria for the study of which 338 (1GTKI n=230, 2GTKI n=108) patients received starting TKI doses consistent with chronic phase CML. Patients initiating therapy with 1GTKI and 2GTKI were generally similar in most baseline characteristics; however, a higher proportion of patients initiating therapy with a 2GTKI were younger, covered by commercial insurance, and using TKI doses consistent with accelerated or blast crisis phase CML (Table 1).

**Table 1.** Sample Characteristics

| | 1GTKI n=237 | 2GTKI n=131 | P-value |
|---|---|---|---|
| Age in years, mean (SD) | 69.9 (11.4) | 67.2 (13.5) | 0.04 |
| Female, n (%) | 125 (52.7%) | 74 (56.5%) | 0.49 |
| Plan type, n (%) | | | |
| Commercial fully-insured | 14 (5.9%) | 17 (13.0%) | 0.04 |
| Medicare Advantage | 94 (39.7%) | 37 (28.2%) | |
| Medicare PDP | 129 (54.4%) | 77 (58.8%) | |
| Geographic region, n (%) | | | |
| Northeast | 15 (6.3%) | 13 (9.9%) | 0.56 |
| Midwest | 50 (21.1%) | 29 (22.1%) | |
| South | 134 (56.5%) | 72 (55.0%) | |
| West | 38 (16.1%) | 17 (13.0%) | |
| Low income subsidy, n (%)* | 54 (24.2%) | 36 (31.6%) | 0.32 |
| Proxy for CML phase, n (%)† | | | |
| Chronic | 230 (97.0%) | 108 (82.4%) | <0.0001 |
| Accelerated or blast crisis | 7 (3.0%) | 23 (17.6%) | |
| $\geq$2 unique concurrent medications‡, n (%) | 133 (56.1%) | 72 (55.0%) | 0.83 |
| Patient monthly out-of-pocket cost for index medication, mean (SD) | $1,566 ($1,204) | $1,844 ($1,586) | 0.12 |
| RxRisk-V Score§, mean (SD) | 5.1 (3.3) | 5.0 (3.1) | 0.69 |
| Oncologist provider, n (%) | 221 (93.3%) | 122 (93.1%) | 0.97 |

1GTKI: first-generation tyrosine kinase inhibitor, 2GTKI: second-generation tyrosine kinase inhibitor, CML: chronic myeloid leukemia, PDP: prescription drug plan, SD: standard deviation

*Low income subsidy only assessed for Medicare patients

†Patients' starting dose of TKI therapy was assessed and used as a proxy for CML phase at the time of initiation

‡ Medication count was assessed at baseline, so TKI therapy was not included as patients were new users. Total number of unique concurrent medications was defined using pharmacy claims data where the service date occurred during the baseline period. The total number of unique concurrent medications was a count of all medications with the exception of TKI therapies. Two or more claims were required to eliminate including medications for short-term use.

§The RxRisk-V score is determined based on the identification of 45 distinct comorbid conditions via their associated medication treatments. A higher score indicates a higher chronic disease burden.[15]

**Figure 2.** Sample Selection

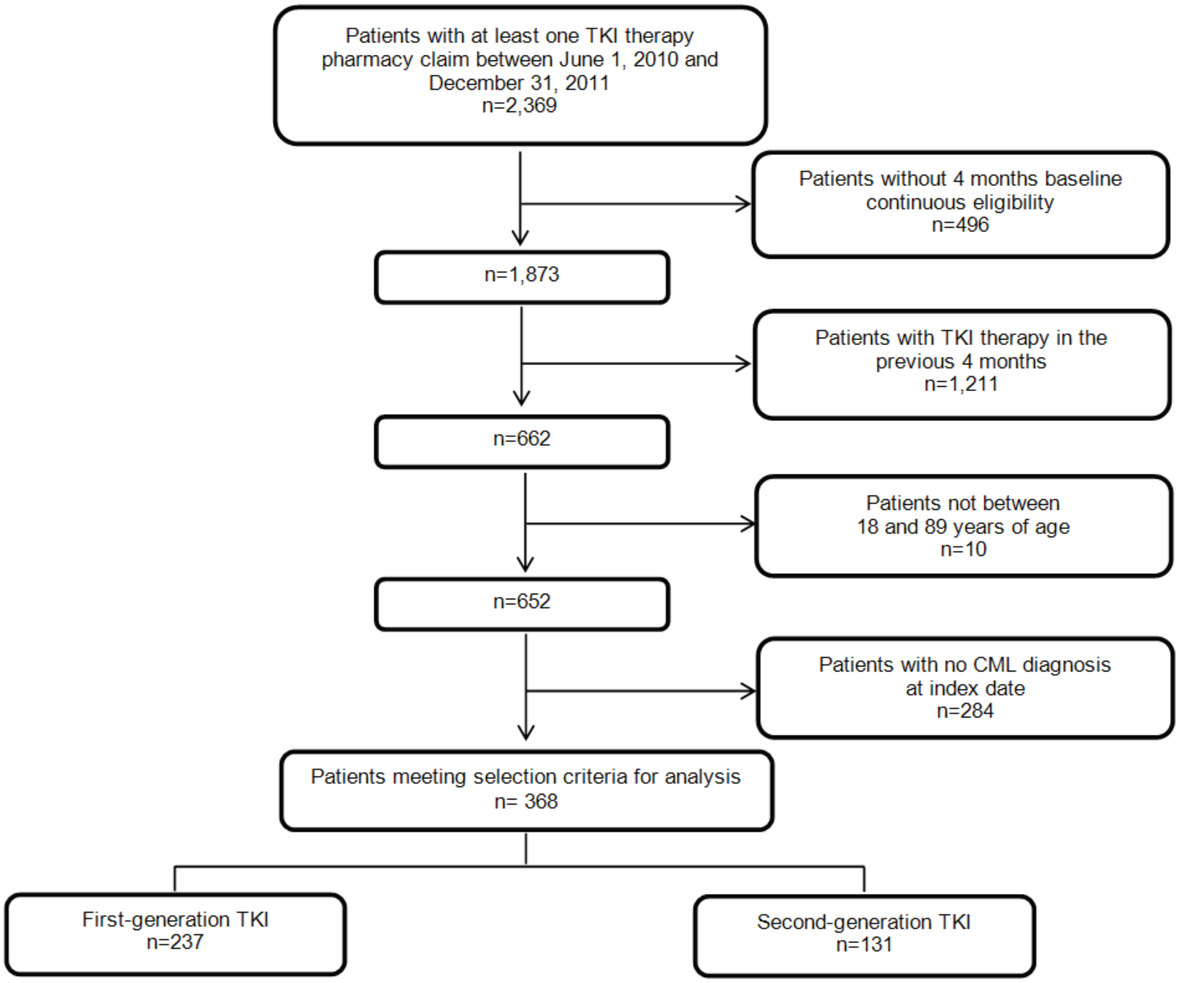

TKI: Tyrosine kinase inhibitor

In the logistic regression model, chronic phase CML and enrollment in a Medicare Advantage plan were the only baseline characteristics significantly associated with initiating a 2GTKI compared to a 1GTKI. Patients with a starting dose that reflected accelerated/blast crisis compared to chronic phase CML were more likely to initiate therapy with a 2GTKI versus 1GTKI (odds ratio [OR]=8.06, 95% confidence interval [CI]: 3.22-20.18]). Patients enrolled in a Medicare Advantage plan (commercial plan was referent) were less likely to initiate a 2GTKI compared to 1GTKI (OR: 0.27, 95% CI: 0.09-0.79).

#### Treatment Interruption

A higher proportion of patients who initiated therapy with a 2GTKI had a treatment interruption compared to patients who initiated therapy with a 1GTKI (P=0.01) (Table 2). Additionally, mean time on therapy was significantly longer for patients initiating a 1GTKI versus 2GTKI (233 and 186 days respectively, P<0.01). After adjusting for covariates, patients receiving a 2GTKI had a 48% higher risk of treatment interruption versus patients receiving a 1GTKI (Figure 3). Two sensitivity analyses were conducted limited to patients initiating therapy with a dose suggestive of chronic phase disease (n=338) and censoring for death as a competing risk (n=368). The results for the sensitivity analyses were consistent with those for the main analyses (chronic phase disease HR: 1.44, 95% CI: 1.03-2.00; censoring for death HR: 1.46, 95% CI: 1.07-1.99).

**Table 2.** Treatment Interruption and Regimen Changes by TKI Treatment Group

| Outcome | 1GTKI n=237 | 2GTKI n=131 | P-value |
|---|---|---|---|
| **Treatment Interruption** | | | |
| n (%) | 107 (45.1%) | 77 (58.8%) | 0.01 |
| Hazard ratio (95% CI) | Reference | 1.48 (1.08-2.02) | 0.01 |
| **Regimen Change** | | | |
| n (%) | 45 (19.0%) | 24 (18.3%) | 0.79 |
| Hazard ratio (95% CI) | Reference | 1.16 (0.68-1.93) | 0.59 |

1GTKI: first-generation tyrosine kinase inhibitor, 2GTKI: second-generation tyrosine kinase inhibitor, CI: confidence interval, HR: hazard ratio

Cox proportional hazard regression models controlled for age, gender, plan type, region, low income subsidy, dual eligibility status, RxRisk-V score, phase of CML disease using starting dose of TKI as a proxy, flu or pneumonia vaccination, medication count, and provider characteristics (age, gender, specialty, provider practice setting).

**Figure 3.** Time to Treatment Interruption (days)

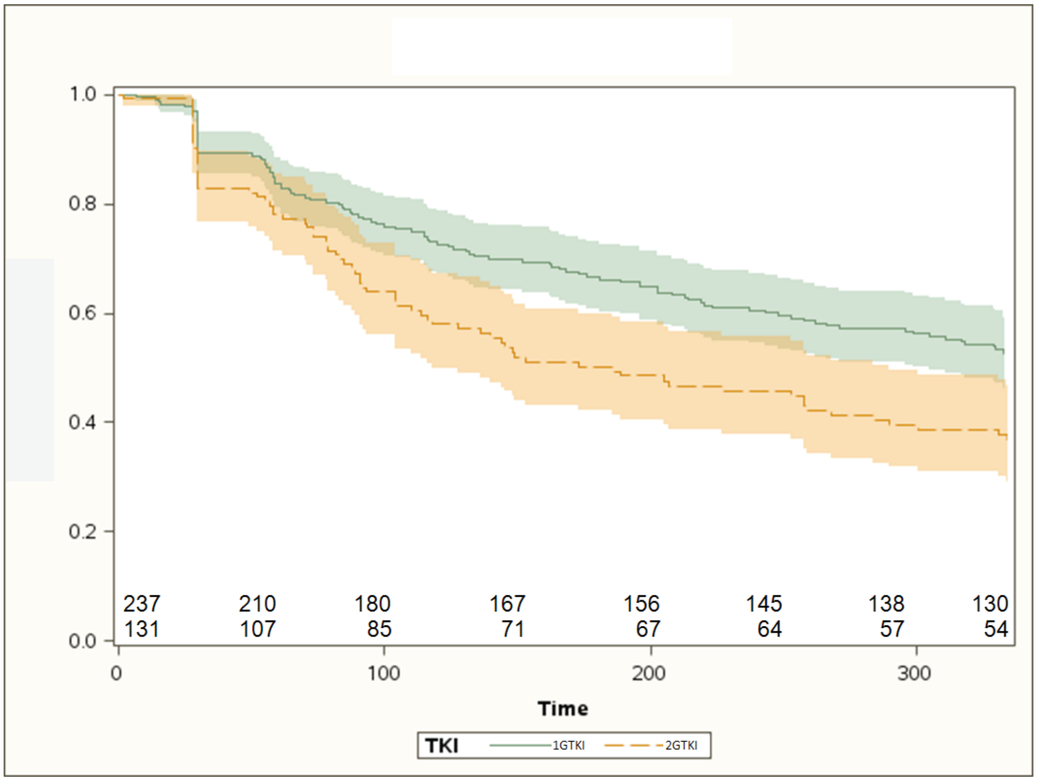

1GTKI: first-generation tyrosine kinase inhibitor, 2GTKI: second-generation tyrosine kinase inhibitor

Cox proportional hazard regression model controlled for age, gender, plan type, region, low income subsidy, dual eligibility status, RxRisk-V score, phase of CML disease using starting dose of TKI as a proxy, flu or pneumonia vaccination, medication count, and provider characteristics (age, gender, specialty, provider practice setting).

Among patients with a treatment interruption, 58% of the 1GTKI initiators re-started the index medication compared to 38% of the 2GTKI initiators. Medication change occurred in 22% of the 1GTKI initiators and 25% of the 2GTKI initiators. Discontinuation of TKI therapy occurred in 15% of the 1GTKI initiators and 30% of the 2GTKI initiators. Less than 9% of patients receiving both treatments dis-enrolled from a Humana plan.

**Regimen Change**

Approximately 19% of patients had a regimen change (Table 2). Regimen changes were more commonly TKI change (13.5% of 1GTKI initiators, 14.5% of 2GTKI initiators) than dose increases (6.3% of 1GTKI initiators, 3.8% of 2GTKI initiators). There were no differences in regimen changes between patients initiating therapy with a 1GTKI and 2GTKI in the unadjusted or adjusted analyses. The results for the sensitivity analysis, limited to patients with chronic phase CML, were consistent with those for the main analyses; there was no difference in TKI regimen change for patients initiating a 2GTKI compared to those initiating therapy with a 1GTKI (HR: 1.13, 95% CI 0.66-1.90).

### DISCUSSION

Our study found that treatment interruptions were significantly more likely in patients initiating therapy with a 2GTKI versus 1GTKI. The proportion of patients with a treatment interruption in our study was similar to that reported in ENESTnd and DASISION at 12 months.[6] Specifically, 59% of patients who initiated therapy with a 2GTKI in our study experienced treatment interruption compared to 59% (300 mg) and 66% (400 mg) of nilotinib patients and 52% of dasatinib patients in Phase III trials.[6-7] For imatinib, we reported 45% treatment interruptions compared to 43% and 35% across the two clinical trials at 12 months.[6-7] Other observational studies reported treatment interruptions of 16% and 31% over at least a 6-month and 12-month follow-up period, respectively.[9-10] Sixteen percent treatment interruptions reported by Saleh et. al. is notably lower than our study and other reports but also had a shorter follow-up period to evaluate this outcome. Additionally, Saleh et. al. used nurses' documentation of treatment interruptions rather than treatment patterns, which may have resulted in lower reports.

We found that approximately 19% of patients had a regimen change during the follow-up period, which most often involved changing to a different TKI. In clinical practice, true indications of treatment failure warrant treatment change including a patient that has lost a CCyR or a complete hematologic response.[11] However, the differences in CCyR and major molecular response (MMR) between 1GTKI and 2GTKI in the clinical trials did not translate to differences in therapy switching between the treatment groups in our observational study.

As expected, a higher proportion of patients with advanced phase disease received a 2GTKI in our study, assessed using initial TKI starting dose as a proxy for CML phase, compared to the imatinib group. Therefore, the observed differences in treatment interruptions and regimen change may be biased by the significant difference in the disease phase of both series of patients. However, sensitivity analyses limited to only patients in chronic phase were conducted and the results were consistent with those for the main analyses.

Our study has several limitations. We did not assess the specific reasons why patients had a treatment interruption or change, as that information was not directly available in the database. Given that the clinical trials are designed to allow treatment interruptions to manage adverse events and that most patients in our study returned to a TKI after the treatment interruption, it is plausible that adverse events may have contributed to treatment interruptions observed in our study. The 2GTKI therapies have unique toxicities which may have contributed to increased treatment interruptions compared to imatinib. Specifically, pleural effusion is a unique adverse event related to dasatinib treatment and QT prolongation has been reported among patients taking nilotinib.[20-21] Additionally, we did not limit eligibility to those patients with medical coverage for this analysis. Therefore, we were not able to identify hospitalizations and bone marrow transplantations due to data limitation (not including medical claims). Hospitalizations and bone marrow transplantations may affect the estimates of treatment interruptions.[9] Also, we cannot rule out residual confounding due to unmeasured and inadequately measured variables. For example, 2GTKIs are more potent and have higher response rates in accelerated and blast crisis; patients with doses indicative of more advanced disease were more likely to receive a 2GTKI. Laboratory results were not available, therefore clinical outcomes such as CCyR, MMR, and disease progression could not be assessed. In addition, the phase of CML was not available in the data, and the starting dose of the index medication was used as a proxy, which has not been validated. However, measured patient and provider characteristics in our study had no impact on initiation of a 2GTKI, which suggested that bias from the residual confounding may be limited. Other limitations common to studies using administrative claims data may apply in this study. These include errors in claims coding and missing data. These errors may not be systematic toward the choice of TKI in our study to confound our study results. Administrative claims data include paid claims only. If a patient were given sample medications, these would not be reflected in the claims data. On the other hand, a claim for a TKI therapy does not necessarily mean the patient actually took the medication.

Despite the limitations of claims data, use of the Humana Research Database permitted a sufficiently large sample size needed to study CML. Because this database includes a large number of people covered by Medicare (aged ≥65 years) and the median age at diagnosis of CML is 64,[22] this database was beneficial for studying CML. However, because this study used claims from a single health plan, it may not be generalizable to the entire population.

In conclusion, this study from a single health plan population found that treatment interruptions were more common among patients initiating therapy with a 2GTKI, yet regimen changes did not vary by generation of TKI. Future research should assess reasons for treatment interruption and investigate these associations in other populations.

### ACKNOWLEDGEMENTS

The authors wish to recognize Tyler Whisman, a clinical pharmacist at Humana Inc. during the time the study was conducted, for his contributions to the concept of this project.

### CONFLICT OF INTEREST DECLARATION

This work was funded by the PhRMA Foundation. The funding organization had no role in the collection, interpretation, or reporting of the data and results. Humana, Inc. provided the data for the research. LH is employed by Humana and MW was employed by Humana at the time the research was conducted. SE is an employee of RTI Health Solutions. GF, CW and SJB have no relevant financial relations to disclose. KR is supported by a Mentored Research Scholar Grant in Applied and Clinical Research (MSRG-12-086-01-TBG) from the American Cancer Society. She is also on the board of The Leukemia & Lymphoma Society (LLS), received travel funding from the American Cancer Society within the past year, and has been an advisory board member for Genentech and received a speaking honorarium from Celgene.
